# Implementación del ADN libre circulante para la detección de aneuploidías fetales

**DOI:** 10.1515/almed-2024-0110

**Published:** 2025-02-28

**Authors:** Irene Madrigal Bajo, Meritxell Jodar Bifet, Celia Badenas Orquin

**Affiliations:** Servicio de Bioquímica y Genética, Hospital Clínic de Barcelona e FCRB-Institut de Investigacions Biomèdiques August Pi I Sunyer (IDIBAPS), Barcelona, España; CIBER de Enfermedades Raras (CIBERER) Barcelona, España; Unidad de Genética, Universitat de Barcelona, Barcelona, España

**Keywords:** ADN libre circulante, cribado prenatal, test prenatal no invasivo, diagnóstico prenatal

## Abstract

**Introducción:**

El ADN libre circulante (ADN-lc) son fragmentos de ADN extracelulares que circulan libremente por la sangre y que se originan a partir de la apoptosis de diversos tipos celulares, como por ejemplo células hematopoyéticas o, en gestaciones, de células trofoblásticas provenientes de la placenta.

**Contenido:**

El estudio del ADN-lc se ha incluido como prueba de cribado prenatal para la detección de anomalías cromosómicas y, a diferencia de otras técnicas invasivas como la amniocentesis o la biopsia de vellosidades coriónicas, se realiza mediante un análisis de sangre materna. Gracias a la utilización de tecnologías avanzadas en el estudio del ADN-lc, como la secuenciación de ADN o los arrays de SNPs, es posible identificar gestaciones con riesgo de trisomía 21, trisomía 18 o trisomía 13.

**Resumen:**

Este test ha demostrado una alta precisión y fiabilidad, con tasas de detección superiores al 99 % para trisomía 21 y una muy baja tasa de falsos positivos y falsos negativos. En algunos países ya se ha implementado como una herramienta de cribado combinado o cribado universal.

**Perspectiva:**

A medida que la tecnología avanza y se vuelve más accesible, se espera que se puedan obtener pruebas aún más precisas para otras anomalías genéticas en el diagnóstico prenatal.

## Introducción

El cribado combinado de primer trimestre (CCPT) y segundo trimestre (CCST) han sido utilizado desde los años 80 para la detección de las aneuploidías fetales más frecuentes como son la trisomía del cromosoma 21 (T21), la trisomía del cromosoma 18 (T18) y la trisomía del cromosoma 13 (T13). A lo largo de los años este cribado ha evolucionado de forma significativa. Actualmente, el CCPT incluye análisis de marcadores bioquímicos en sangre materna como la proteína plasmática asociada al embarazo A (PAPP-A) y la hormona gonadotropina coriónica humana libre beta (β-hCG). El CCST incluye el análisis de niveles de alfa-fetoproteína (AFP), estriol no conjugado (uE3), β-hCG e inhibina A. En ambos casos se incluye además la medición de la translucencia nucal del feto mediante ecografía. La precisión y la capacidad de detección de ambos cribados han mejorado significativamente, reduciendo la tasa de falsos positivos (FP) y minimizando la necesidad de procedimientos invasivos confirmatorios. En los años 90 la tasa de detección (TD) del CCPT era de aproximadamente el 90 %, con una tasa de FP del 5 %. Cuando el CCPT o CCST indican un riesgo alto de aneuploidía, se requiere la realización de una prueba invasiva, ya sea una biopsia de corion o una amniocentesis, que conllevan un pequeño riesgo asociado de aborto. En 1997, Lo y colaboradores descubrieron ADN fetal que circulaba por la sangre materna [1]. Este descubrimiento sentó las bases para el desarrollo de pruebas prenatales no invasivas basadas en el estudio del ADN libre circulante (ADN-lc). En la última década, múltiples estudios han demostrado que el test del ADN-lc fetal en sangre materna permite establecer un riesgo de T13, T18 y T21 con una sensibilidad y especificidad superior al cribado combinado y con una reducción de FP. Esto sugiere que el estudio del ADN-lc podría utilizarse en combinación con el CCPT o de forma universal para mejorar la precisión de la detección de anomalías genéticas en el feto en un estadio temprano del embrazo. El ADN-lc son fragmentos de ADN extracelulares que circulan libremente en el torrente sanguíneo y que puede provenir de diversas fuentes. Todos los individuos presentan ADN-lc en la sangre ya que el origen de este ADN es la apoptosis de diversos tipos celulares, principalmente de células hematopoyéticas [2]. En los últimos años se ha visto que la apoptosis de células procedentes de tumores libera una gran cantidad de ADN al torrente sanguíneo, y su estudio permite realizar un seguimiento de los tumores mediante un test no invasivo [3], 4]. De forma similar se puede realizar un seguimiento de aceptación o rechazo del órgano trasplantado mediante el estudio del ADN liberado al torrente sanguíneo por las células de órganos trasplantados, permitiendo determinar la aceptación o rechazo del órgano trasplantado [5], 6]. En mujeres gestantes existe una mezcla de ADN-lc materno y fetal, y el origen de este último es la apoptosis de células trofoblásticas [7], 8]. El ADN-lc fetal se puede detectar a partir de la semana 6 del embarazo y todo el ADN del feto está representado. Dado que su origen es placentario, su presencia desaparece después del nacimiento. El tamaño está entre 150pb y 200pb, ligeramente inferior al tamaño del ADN-lc materno. Esta diferencia permite, en algunas de las metodologías utilizadas, diferenciar los fragmentos de ADN-lc materno del fetal, y establecer la fracción fetal (FF), es decir qué porcentaje del ADN-lc que se estudia es de origen fetal.

## Metodologías de estudio del ADN-lc en diagnóstico prenatal

La tecnología aplicada para el estudio del ADN-lc ha avanzado de forma significativa en los últimos años y permite la detección de diversas anomalías genéticas de una forma precisa y no invasiva para el feto (Tabla 1).

**Tabla 1:** Principales propiedades de las tecnologías para el estudio de ADN-lc.

Características	NGS	SNPs	RCR
Edad gestacional mínima	10	9	10
Estudio trisomías comunes	SI	SI	SI
Estudio trisomías raras	SI	SI	NO
Estudio anomalías sexuales	SI	SI	SI
Detección triploidía	NO	SI	NO
Detección DUP	NO	SI	NO
Detección CNVs	>7Mb	SI^c^	NO
Detección mosaicos^a^	SI^b^	NO	NO
Estudio gemelares	SI	SI	SI
Determinación FF	SI	SI	NO
Determinación cigosidad	NO	SI	NO

Modificado de Benn et al., 2023. ^a^Descripción página 9. ^b^Si el porcentaje de mosaico es elevado. ^c^Según diseño del array. DUP, disomía uniparental; CNV, variaciones en número de copia; NGS, next generation sequencing; SNP, single nucleotide polymorphisms; RCR, replicación del círculo rodante; FF, fracción fetal.

### Secuenciación masiva

La secuenciación masiva es actualmente una de las más utilizadas en el estudio del ADN-lc, tanto de origen materno como fetal. Esta metodología genera millones de lecturas de secuencias que se alinean a un genoma de referencia humano y posteriormente se contabilizan el número de lecturas para determinar si existe un exceso (trisomía) o déficit (monosomía) de estas secuencias indicando la presencia de aneuploidías de un determinado cromosoma. Esta tecnología permite además ampliar el estudio al resto de cromosomas e identificar variaciones en número de copia (CNV) superiores a 7Mb.

### Análisis de polimorfismos de un solo nucleótido (SNP)

El análisis de polimorfismos de SNP mediante el uso de un array se basa en la proporción relativa de genotipos maternos y fetales para diferenciar entre ambos orígenes [9], 10]. Permite calcular la probabilidad máxima de que el feto presente o no alguna aneuploidía. A diferencia de la secuenciación masiva, es necesario estudiar el plasma (ADN-lc materno y fetal), y ADN materno celular. Este método permite además de la detección de triploidías y disomías uniparentales (DUP) [11], 12].

### Cuantificación directa de fragmentos de ADN-lc o replicación del círculo rodante (RCR)

La RCR es una tecnología que destaca por convertir fragmentos cromosómicos en objetos cuantificables de forma digital, y no requiere amplificación mediante PCR ni secuenciación [13], 14], por lo que esta técnica es económicamente más rentable y accesible. La sensibilidad y especificidad para la detección de aneuploidías es alta, incluso en fracciones fetales más bajas, lo que mejora su solidez y utilidad clínica [15], 16]. Sin embargo, actualmente solo es posible utilizar cuatro fluoróforos (colores) diferentes, lo que limita solo la detección a las aneuploidías más frecuentes (T21, T18 y T13) y aneuploidías de los cromosomas sexuales (ACS).

## Sensibilidad y especificidad en la detección aneuploidías cromosómicas

### Aneuploidías de los cromosomas 21, 18 y 13

El estudio del ADN-lc en sangre materna es una técnica avanzada que permite la detección de T21, T18 y T13 de manera no invasiva para el feto [17]. Este test se puede realizar a partir de semana 10, a partir de la cual el porcentaje de ADN fetal libre circulante puede ser superior al 3 %, permitiendo un mejor control de las gestaciones. Dos grandes estudios han reportado que la frecuencia de detección de T21, T18 y T13 en gestantes con CCPT alto (≥1:200) está ∼2,3 % para T21, de 0,4 % para T18 y de 0,4 % para T13 [18], 19]. En cambio, en gestantes sin seleccionar, la frecuencia de detección se sitúa entre 0,3–0,5 % para T21, 0,07–0.1 % para T18 y el 0,05–0,08 % para T13 [19], 20]. La sensibilidad y especificidad permiten evaluar la precisión y efectividad del estudio del ADN-lc en el contexto del cribado prenatal. Los métodos utilizados para estudiar el ADN-lc varían de forma considerable, sin embargo, existen diferencias relativamente pequeñas en el rendimiento del cribado de las T21, T18 y T13, principalmente en embarazos únicos. La mayoría de los estudios de validación evalúan el rendimiento de la detección de aneuploidías en muestras de plasma materno procedentes de gestaciones en las que se ha realizado un diagnóstico mediante un test invasivo (detección de falsos negativos (FN)) o mediante pacientes nacidos vivos sanos o afectos (detección de FN o FP). La sensibilidad indica la capacidad de la prueba para detectar fetos con aneuploidías específicas. Para la T21, la sensibilidad suele ser muy alta, alrededor del 99.7 % (95 % CI, 99,1–99,9 %). Para la T18 se reduce un poco y está alrededor del 97,8 % (95 % CI 94,9–99,1 %) y para la T13 se sitúa cerca del 97.0 % (95 % CI 65,8–100 %) [19], [21], [22], [23], [24]. La tasa de FP combinada es del 0,13 %, siendo superior en los casos de T18 y T13. Por otro lado, la especificidad indica la capacidad de la prueba para excluir fetos sin aneuploidías específicas, es decir que con un resultado de riesgo bajo por ADN-lc, el feto no presente aneuploidía. En caso de presentarla el resultado sería un FN. Para la detección de aneuploidías comunes la especificidad detectada es superior al 99 % [19], [21], [22], [23], [24]. Los altos porcentajes de sensibilidad y especificidad hacen que el estudio del ADN-lc sea una herramienta fiable y valiosa para la detección temprana de condiciones genéticas como las T21, T18 y T13.

Los valores predictivos positivos (VPP) y negativos (VPN) son métricas esenciales en la evaluación de la precisión y utilidad clínica de las pruebas de ADN-lc en el cribado prenatal. Estos valores dependen de varios factores como pueden ser la prevalencia de la condición, la fracción de ADN-lc fetal, y la sensibilidad y especificidad de la prueba utilizada. En definitiva, estos valores proporcionan información sobre la probabilidad de que un resultado positivo o negativo sea correcto. El VPP es la probabilidad de que un feto presenta aneuploidía cuando el resultado de la prueba es de alto riesgo, y se calcula dividiendo el número de positivos verdaderos por el número de positivos verdaderos más el número de FP. El VPN es la probabilidad de que un feto no presente aneuploidía cuando el resultado de la prueba es de bajo riesgo, y se calcula dividiendo el número de verdaderos negativos por el número de verdaderos negativos más el número de FN. Las técnicas que presentan alta sensibilidad (capacidad para detectar verdaderos positivos) y alta especificidad (capacidad para detectar verdaderos negativos) mejoran ambos valores predictivos. Por ejemplo, el VPP para la T18 puede ser menor que el de la T21 debido a la menor sensibilidad y prevalencia de la T18.

### Aneuploidías del par sexual

Las ACSs incluyen condiciones como el síndrome de Turner (45,X), el síndrome de Klinefelter (47,XXY), el síndrome de Triple X (47,XXX) y otras alteraciones relacionadas con los cromosomas X e Y. En la actualidad, el rendimiento para la detección de ACS es alto [25], 26]. No obstante, este rendimiento puede ser algo inferior en caso de monosomía X, debido fundamentalmente a las tasas más altas de mosaicismo tanto placentario como materno para esta aneuploidía [27], 28]. El mosaicismo es la presencia de células del mismo individuo con diferente dotación genética. La inclusión de ACSs en estudio del ADN-lc ha sido un tema de debate entre los profesionales médicos. Algunas ACSs, como por ejemplo el síndrome de Turner (45,X) o el síndrome Klinefelter (47,XXY) pueden tener repercusiones importantes en la salud o fertilidad, respectivamente. Sin embargo, su inclusión en el estudio del ADN-lc es controvertida ya que existen autores que consideran que estas aneuploidías pueden provocar síntomas leves o ningún síntoma [29] y, si se introducen como prueba de cribado alternativa al cribado combinado, incrementar el coste económico del cribado [30]. Esta variabilidad complica el proceso de toma de decisiones tanto para los obstetras como para los progenitores.

### Aneuploidías cromosómicas raras

La detección de aneuploidías cromosómicas raras (ACR), es decir de cromosomas autosómicos diferentes a los cromosomas 13, 18 y 21, es una herramienta importante a nivel de cribado cromosómico. Si bien la presencia de trisomía completa no es compatible con la vida, su presencia en mosaico, es decir que la trisomía está solo en un porcentaje de células, puede estar vinculada a un funcionamiento anormal de la placenta. Por ejemplo, el mosaicismo placentario de T16 se asocia con problemas en el crecimiento fetal. Estas gestantes presentan un mayor riesgo de preeclampia por lo que debe hacerse un seguimiento más específico del embarazo y probablemente inducir el parto en los casos más graves. Diversos estudios han implementado el análisis de ACR en el estudio del cribado prenatal. La TD de ACR en población general está entre 0,18 % y 0,32 %, con un VPP del 6 %. Las ACR más comunes detectadas son las T7, T8, T16, T20 y T22 [20], 31].

## Embarazos gemelares

La detección de aneuploidías en embarazos gemelares es algo más complejo que en embarazos únicos, pero la sensibilidad y especificidad es similar a la de embarazos únicos [32], 33]. Se debe tener en cuenta que, en embarazos gemelares, el ADN-lc proviene de ambos fetos, lo que puede complicar la interpretación de los resultados. En gemelos monocigóticos (20–30 % gestaciones múltiples) ambos fetos presentan idéntico ADN, por lo que las aneuploidías, en principio, afectan a ambos fetos por igual. En gemelos dicigóticos (60–70 % gestaciones múltiples) es crucial diferenciar entre las contribuciones de cada feto para detectar aneuploidías específicas de cada uno y las diferencias en la contribución de cada feto al ADN-lc total pueden dificultar la identificación de aneuploidías específicas de uno de los fetos. Determinar la FF de cada feto es posible solo con algunas de las técnicas disponibles hoy en día, por lo que la mayoría de las veces no se puede establecer qué feto presenta alto riesgo de la aneuploidía detectada y qué fracción fetal presenta cada uno. Es importante destacar que la presencia de fetos discordantes o gemelos evanescentes puede alterar los resultados del ADN-lc [34]. La incidencia de gemelos evanescentes ocurre aproximadamente en el 50 % de los embarazos con tres o más sacos gestacionales, en el 36 % de los embarazos gemelares y en el 20–30 % de los embarazos logrados con técnicas de reproducción asistida [35].

## Tasa de no resultados

La falta de resultados puede variar ampliamente entre diferentes estudios, pero generalmente se encuentra en el rango del 1 % al 5 %. [12], 36], y en un 50–75 % de los casos, la repetición del análisis con una nueva muestra sanguínea permite obtener un resultado. La baja FF es una de las principales causas en la falta de resultados y, aunque existen discursos contradictorios, puede ser debida a diversos factores, entre los que destacan una placenta pequeña [37], 38] o un índice de masa corporal (IMC) elevado de la gestante posiblemente debido a una mayor inflamación y tejido adiposo necrótico en estas gestantes [39], [40], [41]. En 2018, Chan y colaboradores realizaron un estudio retrospectivo y reportaron que las gestantes con estudios de ADN-lc “sin resultados” tenían un mayor riesgo respecto a un grupo control de presentar aneuploidías cromosómicas (6,5 % versus 0,2 %), preeclampsia (11 % versus 1,5 %) y diabetes gestacional (23 % versus 7,5 %) [42]. Algunas investigaciones sugieren que existe una asociación entre la no obtención de resultados en el estudio del ADN-lc y la presencia de ciertas aneuploidías cromosómicas como el síndrome de Turner o con un mayor riesgo de T13 o T18 [36], 43]. La presencia de enfermedades autoinmunes en la gestante también se ha relacionado con la falta de resultado, principalmente debido a que las reacciones inflamatorias pueden aumentar el porcentaje de ADN-lc materno o enmascarar la presencia del ADN fetal en la sangre materna [44], 45].

## Factores que pueden afectar la sensibilidad y especificidad en el estudio del ADN-lc

Existen diversas circunstancias que pueden alterar el resultado del estudio del ADN-lc, obteniéndose en estos casos resultados discordantes. Diversos estudios han reportado que las causas principales son la presencia de mosaicos fetales o maternos, presencia de gemelos evanescentes o tumores maternos, entre otros [46], 47].

### Presencia de mosaicismos fetales

La detección de mosaicismo es una de las limitaciones descritas en el estudio del ADN-lc. La presencia de mosaicos, tanto placentarios como fetales, puede dar lugar a resultados FP o FN [45]. Aunque se sabe que la información genética de los tejidos placentarios es representativa del feto, en la mayoría de los casos se observa el mosaicismo, principalmente placentario, en 1–2% de los cariotipos [27]. El origen del ADN-lc fetal es placentario mientras que el origen del ADN obtenido del líquido amniótico es fetal, por lo tanto, en gestaciones con mosaicos confinados a placenta, el resultado del ADN-lc y del LA va a ser discordante. En las gestaciones con mosaicos confinados a la placenta el resultado del ADN-lc será FP ya que la aneuploidía solo está presente en un porcentaje de células placentarias. En los mosaicos fetales, el estudio ADN-lc será un FN ya que la aneuploidía está solo presente en un porcentaje de células fetales. Y por último está el mosaicismo generalizado, en el que el mosaico está tanto en placenta como en el feto. El resultado del ADN-lc será concordante con el del feto, siempre y cuando el grado de mosaicismo, es decir porcentaje de células que presentan la aneuploidía, sea parecido en ambos tejidos. Si el grado mosaicismo difiere mucho, es probable obtener resultados discordantes, tanto FP como FN. Recientemente, se ha realizado un estudio para determinar impacto puede tener la presencia de mosaicos en el VPP para la detección de aneuploidías en el estudio del ADN-lc [48]. En este estudio detectaron que el mosaico de T13 era el más común, seguida del mosaico de T18 y del de T21. Además, observaron diferencias significativas en los VPP para las tres trisomías entre muestras con la presencia de mosaicos de bajo porcentaje versus a las de mosaicos de alto porcentaje.

### Presencia de mosaicismos maternos

El mosaicismo materno se refiere a la presencia de dos o más poblaciones de células maternas con diferente composición genética. La presencia de mosaicos maternos puede dar lugar a resultados discordantes. Si una fracción de las células maternas presenta una anomalía cromosómica, podría ser detectada dando la impresión de que esta anomalía está presente en el feto. Un ejemplo es la pérdida gradual y preferencial del cromosoma X inactivado en gestantes con edad avanzada, que resulta en una monosomía X en mosaico en el ADN-lc materno [49], 50].

### Gemelos evanescentes

Los gemelos evanescentes son pérdidas gestacionales que se producen, principalmente durante el primer trimestre de gestación, por la reducción espontánea de uno o más fetos (en caso de gestaciones múltiples). Una causa importante de pérdidas fetales en embarazos múltiples son las aneuploidías cromosómicas. En embarazos gemelares dicigóticos, la FF que aporta cada feto puede diferir, y la presencia de un gemelo evanescente con una alta fracción fetal y discordante con el feto evolutivo, podría complicar la interpretación de los resultados de los estudios del ADN-lc [34], 46], 51], 52].

### Concepciones derivadas de técnicas de fecundación *in vitro* (FIV)

Las concepciones derivadas de FIV presentan placentas generalmente menores que en las concepciones espontáneas, por lo que las FF pueden ser de menor porcentaje [37], 38]. No obstante, algunos estudios no han demostrado diferencias en la FF entre poblaciones de concepciones derivadas de FIV y espontáneas [53], [54], [55].

### Neoplasias maternas

En mujeres gestantes las neoplasias malignas son relativamente raras, pero pueden ocurrir en aproximadamente 1 de cada 1000 casos, siendo los cánceres de mama, melanoma y de cuello uterino los diagnosticados con mayor frecuencia durante el embarazo [56], 57]. Como se ha mencionado anteriormente, el ADN-lc puede proceder también de la apoptosis de células tumorales y la presencia neoplasias maternas puede generar resultados discordantes o no informativos en el ADN-lc [58]. Diversos estudios han reportado la identificación de neoplasias maternas a raíz de los resultados del ADN-lc [59]. De hecho, la detección de múltiples aneuploidías mediante el estudio del ADN-lc podría indicar la presencia de tumores maternos. En estos casos, se debería informar a las gestantes y valorar su derivación a servicios de oncología.

### Trasplante de órganos y transfusiones maternas

Otro factor que puede alterar el resultado del ADN-lc es el trasplante de órganos o médula ósea, que puede generar posibles interferencias en la interpretación de los resultados [60]. Un ejemplo se da en gestantes con trasplantes de donantes masculinos, el ADN-lc podría identificar secuencias del cromosoma Y procedente del órgano trasplantado en gestaciones con feto femenino. En gestantes con trasplantes de médula ósea o de órganos de un donante masculino, se les debe de informar de la posibilidad de tener resultados erróneos y ofrecer pruebas invasivas para la detección de aneuploidías. Por otro lado, si la gestante ha recibido una transfusión sanguínea de donantes masculinos se les recomienda esperar al menos cuatro semanas para realizar el estudio del ADN-lc [61].

### Enfermedades autoinmunes

Diversos estudios han descrito que la FF puede estar alterada debido a diversas reacciones inflamatorias [45], 47], 62]. Por ejemplo, se han informado casos de púrpura trombocitopénica [44], 45] con FF baja, ya que las reacciones inflamatorias pueden aumentar el ADN-lc materno y la proporción de ADN-lc fetal disminuye a medida que aumenta el ADN-lc materno.

## Asesoramiento genético pre-test y post-test

La información al paciente es primordial antes y después de realizar un diagnóstico genético. En este caso, las gestantes deben conocer las ventajas e inconvenientes de cada uno de los test de cribado y de las pruebas invasivas. Es necesaria una correcta formación de los profesionales involucrados en el asesoramiento a las gestantes para que puedan entender adecuadamente las ventajas, limitaciones y los resultados de esta prueba. En algunos países, como por ejemplo España, en función del riesgo de aneuploidía calculado por el CCPT, se recomendará a la gestante la posibilidad de escoger entre realizar un test invasivo o un estudio de ADN-lc. El asesoramiento previo debe informar acerca de los resultados que se pueden obtener, así como de las ventajas y limitaciones de cada prueba. Las pruebas invasivas permiten la detección de otras anomalías genéticas diferentes a las aneuploidías, si bien el motivo de consulta es un CCPT elevado para T21, T18 o T13. Además, estas pruebas permiten la detección de mosaicismos (no inferiores al 15–20 %). Por el contrario, las pruebas invasivas presentan un pequeño riesgo de aborto asociado a la prueba (0,1–0,2 %). Es importante también informar de que un IMC elevado puede relacionarse con la no obtención de resultados. En cuanto a la información post test, se debe informar que un resultado de alto riesgo debe siempre confirmarse con un test invasivo y que un resultado de bajo riesgo no descarta la posibilidad de FN o la presencia de otras anomalías genéticas no estudiadas con el ADN-lc.

## Implementación del ADN-lc en la sanidad pública española y en la comunidad europea

La implementación del estudio del ADN-lc está cambiando los protocolos de cribado en muchos países. La implantación de este estudio permite la detección tanto de aneuploidías cromosómicas y de CNVs, por lo que, en algunos países como Holanda, el CCPT se está sustituyendo por el cribado “no invasivo” en ADN-lc. En España las políticas sanitarias varían en función de cada Comunidad Autónoma, y actualmente existen diferentes protocolos para la aplicación del ADN-lc en el cribado prenatal. En el sistema público, la mayoría de las comunidades autónomas, ofrecen el estudio del ADN-lc a gestantes con CCPT alto o intermedio, tanto en gestaciones únicas como múltiples (máximo 2 fetos). En los casos de riesgo muy alto o la presencia de anomalías ecográficas se recomienda la realización de una técnica invasiva para descartar otro tipo de alteraciones genéticas.

A nivel europeo también existe una falta de consenso para la implementación del estudio del ADN-lc. Actualmente, hay 17 países europeos en los que se ha adoptado este estudio como parte de sus sistemas de atención prenatal financiados con fondos públicos. Los criterios de inclusión varían sustancialmente de un país a otro. En Bélgica y Holanda han eliminado el cribado contingente y se realiza un cribado universal en ADN-lc a todas las gestantes, independientemente del riesgo. Sin embargo, en estos casos no se debería eliminar la ecografía de primer trimestre, ya permite detectar alteraciones en la translucencia nucal, asociadas a otras alteraciones que no se relacionan solo con aneuploidías de T21, T13 y T18. En otros países, como por ejemplo Italia, se realiza un CCPT y en aquellos embarazos con un riesgo determinado se aconseja realizar un test invasivo o el estudio de ADN-lc.

## Beneficios y limitaciones de la implementación del ADN-lc

En el contexto clínico, la implementación del ADN-lc permite la detección temprana (≥10 semanas gestación) de anomalías cromosómicas con una alta sensibilidad y especificidad, y proporciona información crucial para la toma de decisiones tempranas. El estudio del ADN-lc es una prueba robusta y, como se ha mencionado anteriormente, está integrada en el programa de cribado de la mayoría de las Comunidades Autónomas. Esta implementación ha disminuido de forma significativa el número de pruebas invasivas en mujeres con embarazos de alto riesgo, y conlleva una reducción de abortos espontáneos asociados a las pruebas invasivas (0,1–0,2 %), así como la reducción de la ansiedad en los progenitores. Uno de los estudios con mayor número de muestras incluidas es el proyecto TRIDENT. En la primera parte (Trident-1) se evaluó la implementación de pruebas prenatales no invasivas para la detección de aneuploidías después de una prueba de cribado inicial [18], y en la segunda (Trident-2) se evaluó el estudio del ADN-lc como prueba de cribado universal para todas las gestantes [20]. En ambos se concluyó que el estudio del ADN-lc en mujeres con alto riesgo de CCPT (1/200) es más preciso que las pruebas de cribado tradicionales para detectar T21, T18 y T13. El VPP para población general del CCPT fue de 5,2, 4,1 y 1,4 para T21, T18 y T13, respectivamente y el del ADN-lc de 93,5, 80 y 67 para T21, T18 y T13, respectivamente. Además, el ADN-lc presenta una tasa más baja de FP, en comparación con las pruebas de cribado tradicionales [18]. Por otro lado, en Trident-2 concluyeron que el estudio del ADN-lc es una prueba de cribado universal, sin necesidad de realizar una prueba de CCPT previa [20], y que el estudio del ADN-lc es una prueba de cribado robusta para la detección de T21, T18 y T13 y que puede integrarse como programa nacional de cribado de aneuploidías fetales.

En cuanto a la utilidad del estudio del genoma completo versus al dirigido existe todavía algo de controversia, principalmente debido a la relevancia clínica de los hallazgos adicionales o ACR. Recientemente, se analizaron los resultados de seguimiento del estudio Trident-2, para determinar el impacto clínico basado en los resultados del laboratorio y clínica perinatal en aquellos casos en lo que se detectaron ACR (0,36 % de las gestaciones) [63]. La mayoría de las alteraciones cromosómicas fetales (22,1 % de las ACR) fueron patogénicas y asociadas con fenotipos clínicos severos. El 53 % eran mosaicismos placentarios y un 8,5 % de estos se asociaron a preeclampsia y un 13,6 % a un bajo percentil del peso al nacimiento.

En cuanto a las limitaciones, una de las principales es que el estudio del ADN-lc es una prueba de cribado, por lo que los casos con resultado de alto riesgo de aneuploidía deben confirmarse con un test invasivo. Además, no permite la detección de poliploidías (triploidía). La presencia de mosaicos de bajo grado o fetos evanescentes puede provocar resultados discordantes.

## Guías y recomendaciones

Diferentes organizaciones médicas han publicado guías y recomendaciones sobre la implementación del estudio del ADN-lc para la detección de aneuploidías fetales, tanto en embarazos únicos como en embarazos gemelares. En 2023 el Colegio Americano de Genética Médica y Genómica (ACMG) publicó las guías del uso del ADN-lc, recomendando el uso del ADN-lc como cribado universal para la detección de T21, T18, T13 y ACS en gestantes con gestaciones únicas o gemelares [64]. La Sociedad Internacional para el Diagnóstico Prenatal (ISPD) también recomienda el uso del ADN-lc para la detección de las T21, T18 y T13 en gestaciones únicas en gestantes no seleccionadas o gestantes con mayor probabilidad de aneuploidía fetal, y la clasifica también suficientemente precisa para la detección de ACS. Estas sociedades concluyen que el estudio del ADN-lc es suficientemente sensible para ofrecer la prueba como cribado de detección primaria tanto universal como contingente [65].

En cuanto al uso de la detección ACR existe una controversia sobre su aplicación. Las guías profesionales no recomiendan su uso debido a que la evidencia es insuficiente sobre su validez clínica. Para las ACR (en mosaico) el VPP es bajo, pero en algunas es similar al de la T13 [66]. Además, el efecto sobre el desarrollo fetal de estas ACR puede ser al menos tan grave como la T21 y puede estar asociado con resultados perinatales adversos [67], 68].

## Conclusiones

La implementación del ADN-lc en la práctica clínica ha transformado la medicina prenatal, permitiendo la detección temprana de aneuploidías fetales en muestras de sangre materna, con una alta sensibilidad y especificidad y reduciendo de forma significativa el número de procedimientos invasivos. Actualmente este cribado se usa de forma combinada o universal para la detección de aneuploidías fetales, pero también se ha empezado a implantar para otras finalidades, como puede ser la detección de mutaciones puntuales responsables de enfermedades, como la acondroplasia o la fibrosis quística. Sin embargo, todavía existen algunas limitaciones para detectar otro tipo anomalías genéticas, especialmente en lo que respecta a la realización de un exoma prenatal utilizando muestras de ADN-lc. El futuro de las pruebas prenatales no invasivas es prometedor para la medicina fetal. Se espera que con los avances tecnológicos y aplicaciones emergentes se puedan obtener pruebas aún más precisas en el diagnóstico prenatal.
